# Changes in Bioactive Compounds, Antioxidant Activity, and Nutritional Quality of Blood Orange Cultivars at Different Storage Temperatures

**DOI:** 10.3390/antiox9101016

**Published:** 2020-10-20

**Authors:** Fariborz Habibi, Asghar Ramezanian, Fabián Guillén, Salvador Castillo, María Serrano, Daniel Valero

**Affiliations:** 1 Department of Horticultural Science, School of Agriculture, Shiraz University, Shiraz 71441-65186, Iran; fariborz_h659@yahoo.com (F.H.); ramezanian@shirazu.ac.ir (A.R.); 2 Department of Food Technology, University Miguel Hernández. Ctra. Beniel km. 3.2, 03312 Orihuela, Alicante, Spain; fabian.guillen@umh.es (F.G.); scastillo@umh.es (S.C.); 3 Department of Applied Biology, University Miguel Hernández. Ctra. Beniel km. 3.2, 03312 Orihuela, Alicante, Spain; m.serrano@umh.es

**Keywords:** organic acids, sugars, anthocyanins, antioxidant enzymes, ascorbic acid

## Abstract

Information about the postharvest physiological behavior of blood orange cultivars can provide comprehensive insight into the best period of storage to maintain the highest fruit quality during prolonged cold storage. In this paper, changes in nutritional quality, bioactive compounds, and antioxidant enzymes in the juice of four blood orange cultivars (“Moro”, “Tarocco”, “Sanguinello”, and “Sanguine”) stored at 2 and 5 °C were studied. Parameters were measured after 0, 30, 60, 90, 120, 150, and 180 days, plus 2 days at 20 °C for shelf life. Sucrose was the sugar found in higher concentrations and decreased during storage in all cultivars, as did glucose and fructose. Organic acids decreased at both temperatures, with the highest content found in “Sanguinello”, especially major (citric acid) and ascorbic acid. Total phenolics content (TPC), total anthocyanins (TAC), and individual cyanidin 3-glucoside and cyanidin 3-(6″-malonylglucoside) increased for all cultivars, with “Sanguinello” having higher concentrations. The antioxidant enzymes catalase (CAT), ascorbate peroxidase (APX), and superoxide dismutase (SOD) were also higher in “Sanguinello” and increased during storage. Overall, these results together with the sensory analysis suggested that “Sanguinello” would be the best cultivar for prolonged storage. The results of this study could be useful to select the best storage duration and temperature for each cultivar and provide the presence of such a high-value commodity for fresh consumption or juice processing long after the harvest season.

## 1. Introduction

Blood oranges (*Citrus sinensis* L. Osbeck) are one of four groups within the sweet orange species. The main difference between blood orange fruit and other oranges is the synthesis of anthocyanins pigment in the flesh and sometimes in the peel [1]. The most important commercial cultivars of blood oranges are “Moro”, “Tarocco”, “Sanguinello”, and “Sanguine” [2], which are the results of spontaneous bud mutations [3]. Blood oranges are consumed as fresh fruit due to being a rich source of bioactive compounds such as anthocyanins, ascorbic acid, hydroxycinnamic acids, and flavonoids [4]. Anthocyanin content is considered an important quality component due to the attributed antioxidant activity of these compounds. The antioxidant activity of anthocyanin in blood orange fruits is useful for human health, with an impact on some diseases, including anti-inflammatory, anticancer, and antidiabetic, due to the prevention of oxidation and free-radical chain reactions [5,6,7,8,9]. In blood oranges, 10 anthocyanins were previously identified [6,10], with the major anthocyanins in the flesh being cyanidin 3-glucoside and cyanidin 3-(6″-malonylglucoside) [1,11].

Blood orange cultivars exhibit different levels of pigmentation under similar growing conditions. For example, “Moro” has the highest pigmentation, followed by “Sanguinello” and “Tarocco”; therefore, genetic background can be considered as the main factor accounting for a range of anthocyanin levels [12]. Besides cultivar type, factors including cultural practices, soil characteristics, region of cultivation, climate conditions, environmental conditions, physiological factors, maturity stage, and harvesting time can affect anthocyanin accumulation in blood oranges [3,12]. In addition, blood oranges need a wide day/night temperature range to obtain high anthocyanin concentrations in the flesh [13]; in subtropical or tropical climates, commercial production is limited due to very low anthocyanin concentrations during commercial maturity [11].

Blood oranges are exceptional fruit since, unlike non-climacteric fruit, they can increase in internal quality after harvest by synthesis of anthocyanins during cold storage [1]. It has been reported that cold temperatures below 6 °C can induce anthocyanin synthesis in blood oranges during storage due to the activation of enzymes involved in the phenylpropanoid pathway, including phenylalanine ammonia-lyase (PAL), chalcone synthase (CHS), dihydroflavonol 4-reductase (DFR), and uridine diphosphate (UDP)-glucose flavonoid glucosyl transferase (UFGT) [14]. Therefore, cold storage can be used as a simple technology to enhance anthocyanin accumulation in blood oranges that are poorly pigmented in the aforementioned climates [15].

Low temperature is one of the most effective methods of maintaining the quality of blood orange fruit and can reduce the respiration rate and decay of fruit, while also increasing bioactive compounds [16]. Storage of blood orange fruit at relatively high temperatures can deteriorate bioactive compounds and rapidly decrease shelf life due to water loss, weight loss, high respiration, senescence, and fungal decay [1]. To prevent these problems, storage of blood orange fruit at low temperatures is recommended to maintain these bioactive compounds and to extend postharvest life [1,16].

Since blood orange cultivars have distinct pomological, physiological, and biochemical characteristics, consequently, it is possible to find different storability and postharvest physiological behaviors. Several works have shown a relationship between anthocyanin synthesis in blood oranges and low–moderate storage temperature [11,15,16,17]. However, information about the prolonged storage of blood orange cultivars stored at temperatures below 6 °C is currently poorly understood. Additionally, changes in bioactive compounds, antioxidant activity, and nutritional quality of blood orange can give very useful information for finding the best storage period and preservation to achieve the highest quality of each cultivar for the market. Further, based on the cold-dependency of blood orange fruits for anthocyanin synthesis, the comparison of two low temperatures and the efficiency of lower temperatures (below 3 °C) to enhance anthocyanin accumulation remain unclear. Therefore, the objective of this study was to compare the behavior of four blood orange cultivars (“Moro”, “Tarocco”, “Sanguinello”, and “Sanguine”) stored at 2 and 5 °C for 180 days by establishing (1) the optimal storage period to maintain higher contents of bioactive compounds (total phenolics, total anthocyanins, and ascorbic acid), (2) the efficiency of temperature to enhance major individual anthocyanin concentrations, (3) the response of sugars and organic acids in each cultivar to cold temperatures, and (4) the maximum storability of the cultivars at the two temperatures.

## 2. Materials and Methods

### 2.1. Plant Materials and Storage Conditions

Cultivars of blood oranges, “Moro”, “Tarocco”, “Sanguinello”, and “Sanguine”, were harvested from the commercial citrus orchard of Dashtenaz company in Sari (36.5659° N, 53.0586° E), Mazandaran province, Iran, in mid-January 2018 and immediately transported to the postharvest laboratory. In this area of Iran, the climate is subtropical; the four cultivars reached commercial maturity in January and were harvested at the same time according to the total soluble solids (TSS) and titratable acidity (TA) ratio. The trees were seven years old and grafted on “C-35” citrange (*Citrus sinensis* L. Osbeck × *Poncirus trifoliata* L. Raf.) rootstock. All trees were grown under the same conditions and cultural practices. Fruit were selected based on uniformity of size, checked for no defects or rind injuries, disinfected with 2% sodium hypochlorite (NaOCl) solution, and rinsed with distilled water. Fruit were divided into sets of three replicates with five fruit in each replicate, placed in a polyethylene bag containing 16 holes, and stored for 180 days at 2 and 5 °C and 90% relative humidity (RH). Parameters were measured after 0, 30, 60, 90, 120, 150, and 180 days of cold storage, plus 2 days at 20 °C (shelf life). For each cultivar, sampling date, and replicate, the 5 fruits were cut into two halves for a total of 10 halves. Then, 5 halves were squeezed and the juice was extracted to be used for total phenolics, total anthocyanins, and antioxidant activity determinations, and the other halves were freeze-dried (FD-5003-BT, Iran) for measurement of individual sugars and organic acids, individual anthocyanins, and antioxidant enzyme activities in the juice.

### 2.2. Bioactive Compounds and Antioxidant Activity

The content of total anthocyanin (TAC) was evaluated using the pH differential method previously reported [15]. In brief, potassium chloride (KCl, pH 1.0) and sodium acetate (C_2_H_3_NaO_2_) buffer at pH 4.5 were added to the obtained juice (1:4 dilution) and the absorbance read at 510 and 700 nm (Epoch microplate spectrophotometer, USA) and TAC was calculated as mg L^−1^. The molecular weight and molar extinction coefficient of cyanidin-3-glucoside were 449.2 and 26,900, respectively. For measuring the concentration of total phenolics (TPC), the Folin–Ciocalteu method was used. A juice aliquot (32 µL) was diluted with 2% Na_2_CO_3_ (sodium carbonate, 900 µl) and mixed for 3 min, and then, the Folin reagent was deposited in each tube and kept in darkness for 30 min. Different concentrations of gallic acid were used for preparation of the standard curve. Samples and the standard were measured at 750 nm and the results were reported as mg gallic acid equivalents (GAE) L^−1^ [18].

The determination of total antioxidant activity (TAA) was based on the protocol of Brand-Williams et al. [19]. Briefly, juice (100 µL) was mixed with 0.1 mM 2,2-diphenyl-1-picrylhydrazyl (DPPH, 1 mL) and Tris-HCl buffer (1 mL, pH = 7.5) and kept for 30 min in obscurity, and then, the absorbance reading (517 nm) and TAA were calculated as a percentage with the following equation:

(1) $$\mathrm{TAA}\;\left(\%\right)\;=\;[1\;-\;\frac{\mathrm{Absorbance}\;\mathrm{of}\;\mathrm{sample}\;}{\mathrm{Absorbance}\;\mathrm{of}\;\mathrm{control}}]\;\times \;100$$

### 2.3. High Performance Liquid Chromatography (HPLC) Analyses

#### 2.3.1. Individual Anthocyanin

For individual anthocyanins, the protocol of Martínez-Esplá et al. [20] was adapted to our conditions. Freeze-dried (0.5 g) tissue was pulverized and homogenized with 10 mL solution containing methanol/formic acid/water (79:1:20, *v/v/v*). After centrifugation (10 min at 10,500× *g*), the upper phase was subjected to filtration (0.45 µm filter) and an aliquot of 20 µL sample injected into a HPLC. The individual anthocyanins were separated in a 25 × 0.46 cm Luna C18 column equipped with a C18 guard pre-column (Phenomenex, Macclesfield, UK). The concentrations of both anthocyanins (cyanidin 3-glucoside and cyanidin 3-(6″-malonylglucoside)) were calculated (mg L^−1^) after detection at 520 nm and compared with known standards. HPLC chromatograms of individual anthocyanins are presented in Figure S1. The precision and recovery of HPLC data for individual anthocyanins are presented in Table S1.

#### 2.3.2. Individual Sugars and Organic Acids

In order to quantify organic acids and sugars, the method described by Martínez-Esplá et al. [20] was used. Briefly, 10 mL phosphate buffer (50 mmol L^−1^, pH = 7.8) was added to 0.5 g of freeze-dried tissue, and after homogenization and centrifugation (at 10,500× *g* for 10 min), 10 µL of sample was injected into HPLC (Hewlett-Packard HPLC Series 1100) after filtration by using a 0.45 µm filter. The mobile phase was 0.1% phosphoric acid under isocratic conditions at 0.5 mL min^−1^ rate. For organic acids determination, the absorbance detector was set at 210 nm (UV), while sugars were detected by refractive index. HPLC chromatograms of individual sugars are presented in Figure S2. The precision and recovery of HPLC data for individual sugars are presented in Table S2. Individual organic acids were quantified by using standard curves (citric, ascorbic, malic, oxalic, and succinic acids) and calculated as mg 100 g^−1^, with the exception of citric acid (g 100 g^−1^). HPLC chromatograms of individual organic acids are presented in Figure S3. The precision and recovery of HPLC data for individual organic acids are presented in Table S3. Standard curves of pure sugars (sucrose, glucose, and fructose) were used to quantify the individual sugars and were expressed as g 100 g^−1^; both standards were purchased from Sigma-Aldrich (Sigma-Aldrich, Madrid, Spain).

### 2.4. Enzyme Activities Assay

The protocol for enzyme activities was the same that for flavedo tissue [21]. In brief, the enzyme activities of flesh were evaluated spectrophotometrically. The activities of catalase (CAT) and peroxidase (POD) were determined at 240 and 470 nm, respectively, based on the Chance and Maehly method [22]. Ascorbate peroxidase (APX) activity was assessed at 290 nm [23]. Superoxide dismutase (SOD) activity was measured at 560 nm by the Beauchamp and Fridovich method [24]. In order to assay phenylalanine ammonia-lyase (PAL) activity, the method described by Liu et al. [25] was used. Polyphenol oxidase (PPO) activity was measured at 425 nm [26]. Total protein content of the enzymes extract was measured according to the Bradford method [27]. Briefly, 100 mg of Coomassie Brilliant Blue G-250 was weighed and then dissolved in 50 mL of 95% ethanol. Then, 100 mL of 85% orthophosphoric acid (H_3_PO_4_) was added to the aforesaid solution and the volume reached to 1000 mL with distilled water. Then, 5 mL of Bradford reagent and 100 µL protein extraction were added in the test tube and shaken vigorously for a few seconds. The protein extraction was the same as the antioxidant enzyme samples. The reaction mixture was read using a spectrophotometer at 595 nm. Bovine serum albumin (BSA) was used to elaborate a standard curve [27]. The results for all specific enzyme activities were expressed as U mg^−1^ protein.

### 2.5. Sensory Quality Evaluation

Blood orange cultivars were submitted to descriptive sensory analysis for market acceptability by using ten trained panelists (five men and five women). In this case, fruit was peeled with a knife and segments were separated with hand, then placed in glass dishes with three-digit codes. Each combination of segments prepared from five fruit of three replicates from each cultivar and 3 scattered segments of fruit were tasted by the panelists. For each cultivar, the edible quality was evaluated by a 9-point scale, in which 1 was the poorest and 9 the maximum edible quality, in accordance with the method previously reported [28].

### 2.6. Statistical Analysis

A totally randomized experiment was conducted according to a completely randomized design (CRD) by using three replicates for each cultivar. Data were subjected to analysis of variance (ANOVA) based on three factors (cultivar, temperature, and storage time). Mean values with standard errors (SE) of the means were separated by least significant difference (LSD test, *p* < 0.05) by using the SAS software package v. 9.4 for Windows. Linear regressions were performed with SigmaPlot software v. 11.

## 3. Results

### 3.1. Sugars and Organic Acids

**Figure 1:**
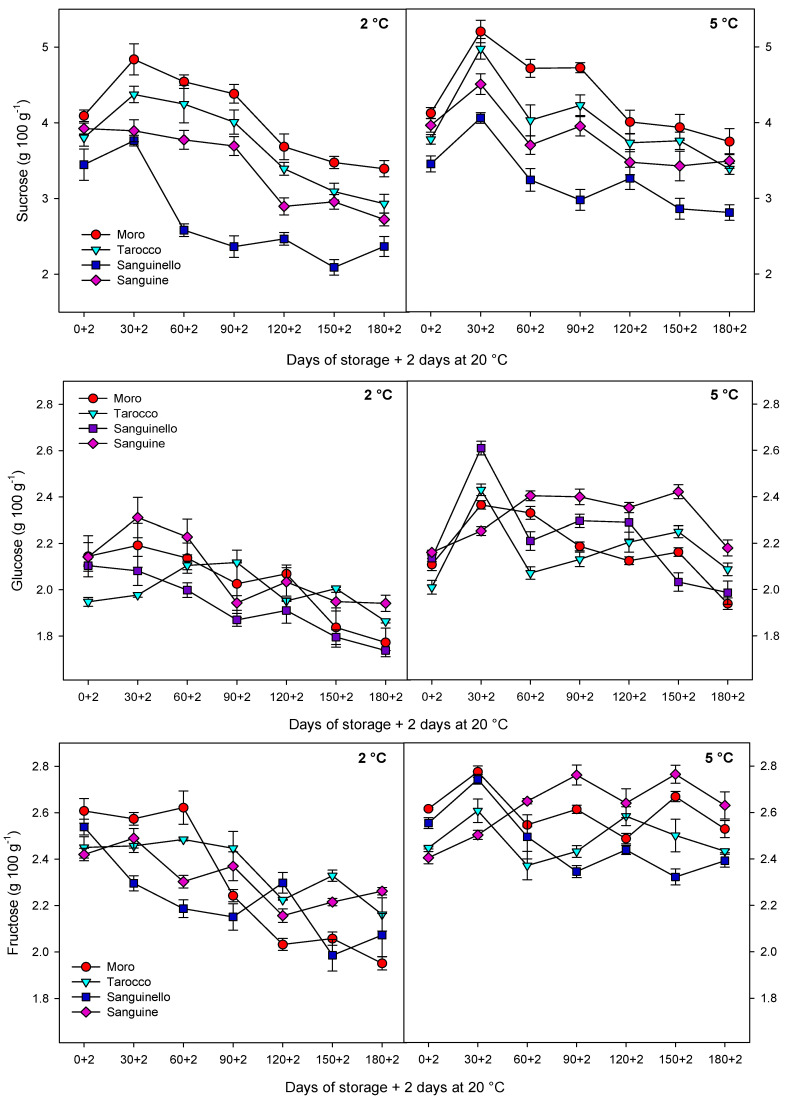
Changes in individual sugars sucrose, glucose, and fructose of blood orange cultivars (“Moro”, “Tarocco”, “Sanguinello”, and “Sanguine”) stored at 2 and 5 °C for 180 days plus 2 days at 20 °C. Vertical bars represent ± standard error (SE) of means. Least significant difference (LSD, *p* < 0.05) values are 0.41, 0.21, and 0.37, for sucrose, glucose, and fructose, respectively.

Individual sugars (sucrose, glucose, and fructose) were quantified by HPLC-refractive index detection (Figure S2) and were affected in all cultivars during storage at both temperatures (Figure 1). Sucrose, glucose, and fructose at 5 °C were 6.73%, 9.54%, and 9.63% higher than 2 °C, respectively. The highest sucrose concentration was found in the “Moro” and “Tarocco” cultivars at both temperatures, and the lowest concentrations of sucrose, glucose, and fructose were observed in “Sanguinello”. Sucrose increased in all cultivars and then decreased towards the end of storage at both temperatures. With respect to glucose and fructose, these sugars diminished in all cultivars, especially at 2 °C. “Sanguinello” showed the lowest levels of both sugars at the end of storage either at 2 or 5 °C.

**Figure 2:**
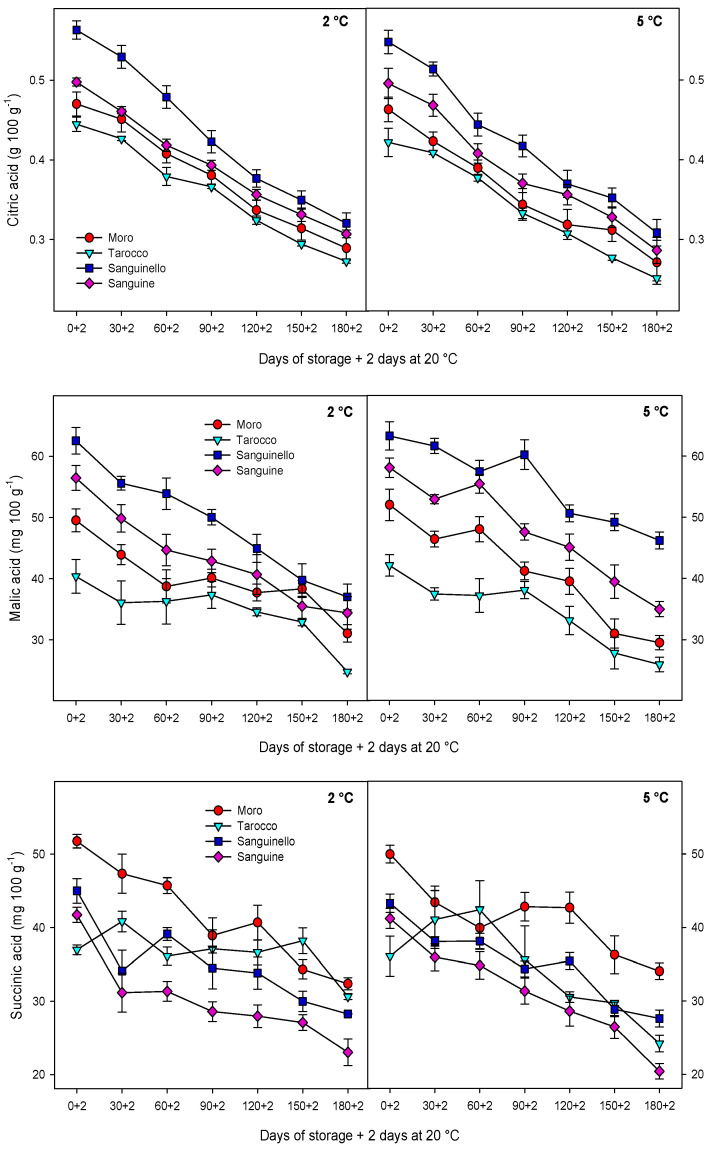

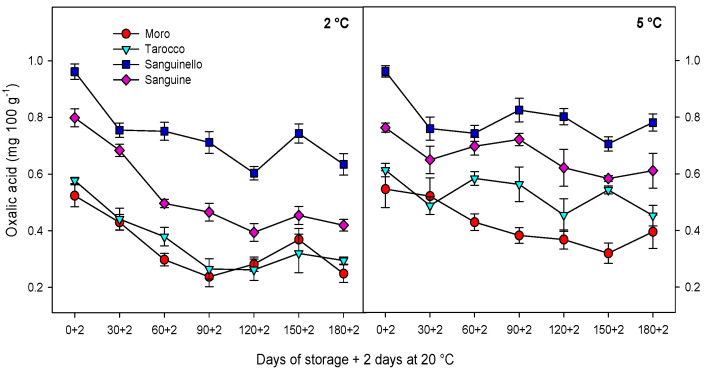
Changes in individual organic acids: citric, malic, succinic, and oxalic acids of blood orange cultivars (“Moro”, “Tarocco”, “Sanguinello”, and “Sanguine”) stored at 2 and 5 °C for 180 days plus 2 days at 20 °C. Vertical bars represent ± standard error (SE) of means. LSD (*p* < 0.05) values 0.05, 4.11, 5.98, and 0.09, for citric, malic, succinic, and oxalic acids, respectively.

Individual organic acids (citric acid, malic acid, succinic acid, and oxalic acid) were quantified by HPLC-diode-array detector (HPLC-DAD) detection (Figure S3) of blood orange cultivars significantly and were affected during storage at both temperatures. The major organic acid was citric acid, followed by malic and succinic acids, and the lowest was oxalic acid (Figure 2). 

### 3.2. Bioactive Compounds and Antioxidant Activity

**Figure 3:**
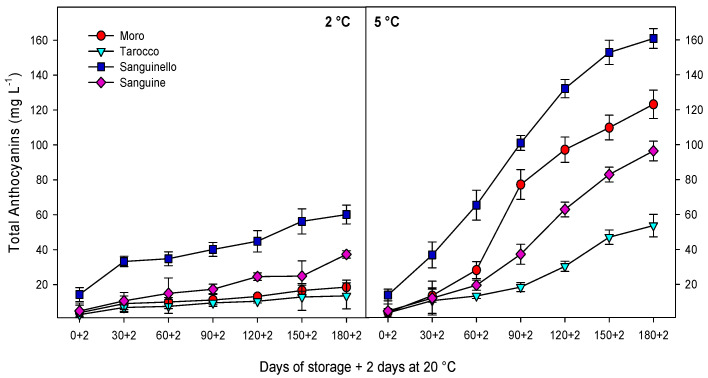

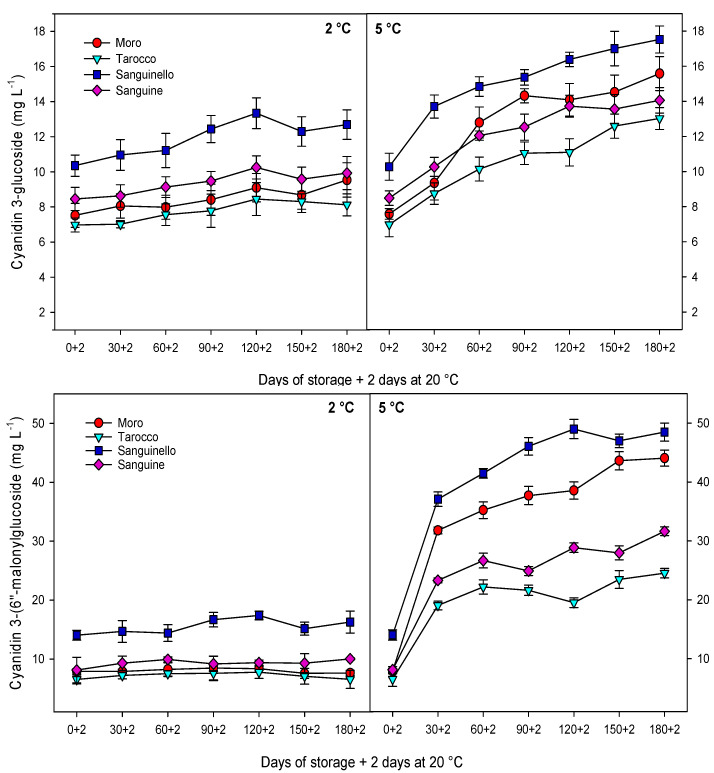
Changes in total anthocyanin concentration, cyanidin-3-glycoside, and cyanidin 3-(6″-malonylglucoside) of blood orange cultivars (“Moro”, “Tarocco”, “Sanguinello”, and “Sanguine”) stored at 2 and 5 °C for 180 days plus 2 days at 20 °C. Vertical bars represent ± standard error (SE) of means. LSD (*p* < 0.05) values are 3.2, 1.7, and 2.4, for total anthocyanin concentration, cyanidin-3-glycoside, and cyanidin 3-(6″-malonylglucoside), respectively.

Figure 3 shows total anthocyanin concentration (TAC), while the major individual anthocyanins are shown in Figure S1 by HPLC-UV–visible determination. For all cultivars, a significant increase in TAC was observed during cold storage at both temperatures, the increase being enhanced at 5 °C (65% higher). Among cultivars, “Sanguinello” reached the highest TAC (160.8 ± 5.6 mg L^−1^), while “Tarocco” showed the lowest (53.6 ± 6.4 mg L^−1^) at the end of storage at 5 °C. Two major individual anthocyanins were detected by HPLC-DAD for all cultivars: cyanidin 3-glucoside and cyanidin 3-(6″-malonylglucoside), which was found at higher concentration. For both, and similarly to TAC, levels were higher at 5 than at 2 °C and “Sanguinello” reached the maximum contents for both cyanidin 3-glucoside and cyanidin 3-(6″-malonylglucoside), while “Tarocco” had the lowest levels of these anthocyanins.

**Figure 4:**
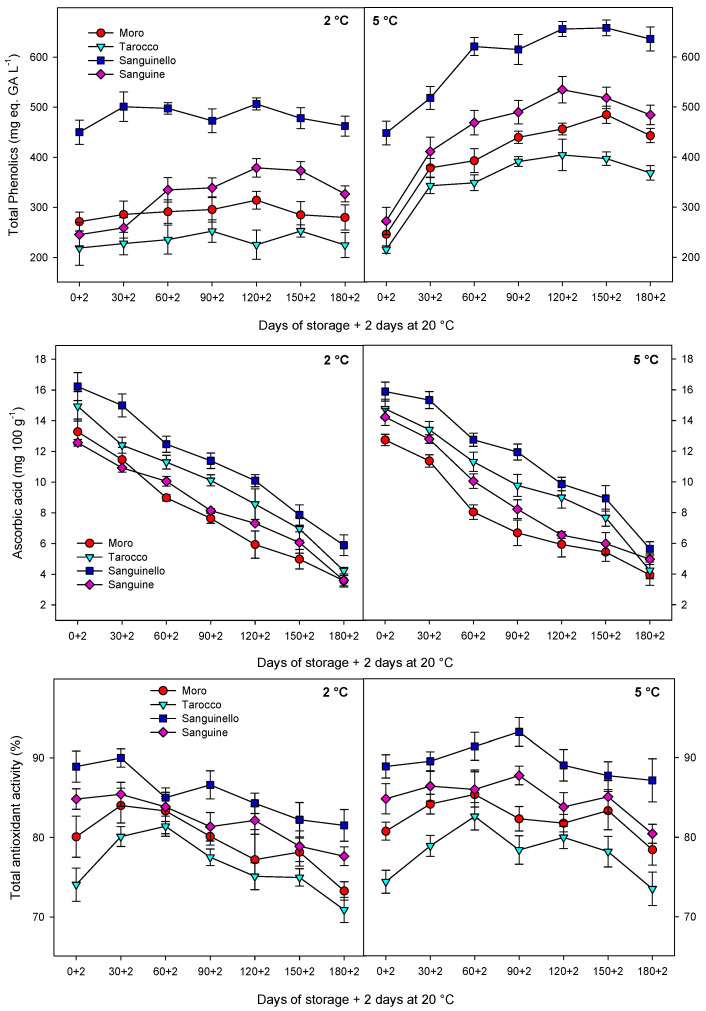
Changes in total phenolics, ascorbic acid, and total antioxidant activity of blood orange cultivars (“Moro”, “Tarocco”, “Sanguinello”, and “Sanguine”) stored at 2 and 5 °C for 180 days plus 2 days at 20 °C. Vertical bars represent ± standard error (SE) of means. LSD (*p* < 0.05) values are 31.55, 0.44, and 4.29, for total phenolics, ascorbic acid, and total antioxidant activity, respectively.

Total phenolic content (TPC) was affected by cultivars, storage, and temperatures (Figure 4). TPC increased for all cultivars and then remained constant or decreased to the end of storage at both temperatures, the increase being enhanced at 5 °C (27% higher). Among cultivars, the highest and the lowest TPC were observed for “Sanguinello” and “Tarocco” with concentrations of 636 ± 24 and 368 ± 14 mg eq. of gallic acid L^−1^, respectively, at the end of storage. 

Ascorbic acid content at harvest was different among cultivars, with “Sanguinello” and “Moro” having the highest and lowest concentrations, respectively (Figure 4). For all, ascorbic acid concentration was significantly reduced during storage at both 2 and 5 °C, although the final levels were higher in “Sanguinello” at 2 (5.89 ± 0.68 mg 100 g^−1^) and 5 °C (5.64 ± 0.46 mg 100 g^−1^), respectively.

Total antioxidant activity (TAA) increased for all cultivars and then decreased towards the end of storage at both temperatures (Figure 4), although TAA at 5 °C was higher than 2 °C. In addition, the reduction in TAA at 2 °C was greater than 5 °C during storage. The highest TAA was observed in “Sanguinello” throughout storage at both temperatures and the lowest TAA was shown in “Tarocco”.

### 3.3. Enzyme Activities

**Figure 5:**
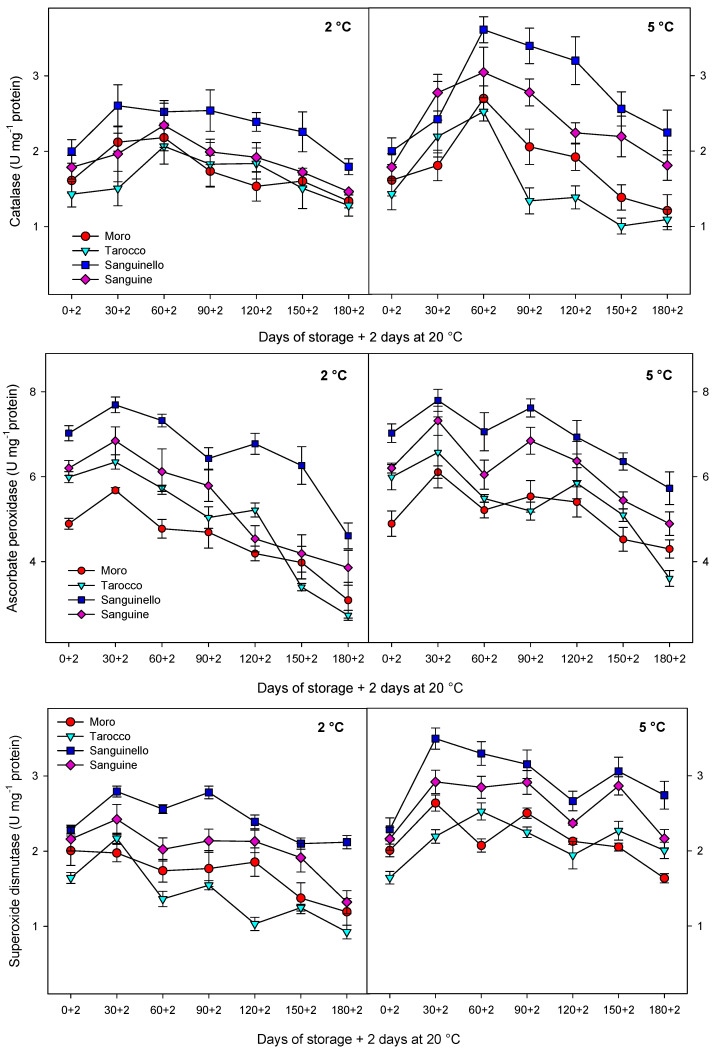
Changes in catalase (CAT), ascorbate peroxidase (APX), and superoxide dismutase (SOD) enzyme activities of blood orange cultivars (“Moro”, “Tarocco”, “Sanguinello”, and “Sanguine”) stored at 2 and 5 °C for 180 days plus 2 days at 20 °C. Vertical bars represent ± standard error (SE) of means. LSD (*p* < 0.05) values are 0.69, 1.04, and 0.30, for CAT, APX, and SOD, respectively.

The activities of the antioxidant enzymes (CAT, APX, and SOD) in the flesh were affected by cultivars, storage times, and temperatures (Figure 5), although POD was not found in any cultivars. For all, the activity was higher at 5 than at 2 °C (12%, 10%, and 23% higher for CAT, APX, and SOD, respectively).

The activities of the antioxidant enzymes increased up to 30 or 60 days and then, decreased towards the end of storage at both temperatures, this reduction being greater at 2 than 5 °C. Among cultivars, “Sanguinello” had the highest level of antioxidant enzyme activities at both temperatures, while “Moro” and “Tarocco” showed lower antioxidant enzyme activities at both storage conditions.

**Figure 6:**
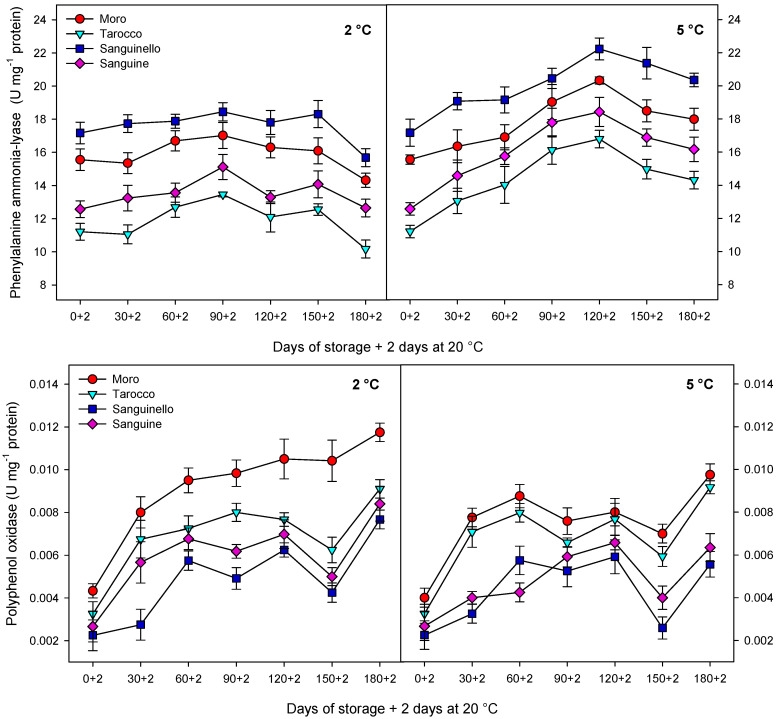
Changes in phenylalanine ammonia-lyase (PAL) and polyphenol oxidase (PPO) enzyme activities of blood orange cultivars (“Moro”, “Tarocco”, “Sanguinello”, and “Sanguine”) stored at 2 and 5 °C for 180 days plus 2 days at 20 °C. Vertical bars represent ± standard error (SE) of means. LSD (*p* < 0.05) values are 1.91 and 0.005 for PAL and PPO, respectively.

PAL activity in the flesh of the cultivars was affected at both temperatures during storage (Figure 6). PAL activity increased up to 120 days in all cultivars at 5 °C and then, decreased to the end of storage. Among cultivars, “Sanguinello” showed the highest PAL activity and “Tarocco” the lowest during storage at both temperatures, although PAL activity at 5 °C was 14% higher than at 2 °C. PPO activity increased during storage at both temperatures, although the activity was very low for all cultivars during cold storage (Figure 6). “Moro” had the highest PPO activity during cold storage, especially at the lowest temperature.

### 3.4. Sensory Quality

Acceptability of the blood orange cultivars was analyzed during storage at both temperatures. The highest edible quality was scored immediately after harvest (9 points for both 2 and 5 °C). For all cultivars, edible quality was always higher at 5 than at 2 °C, although it decreased at both temperatures. At the end of storage, “Moro” and “Tarocco” showed the lower scores at 2 °C (3.1 ± 0.12 and 2.1 ± 0.15, respectively). “Sanguinello” was evaluated as the best cultivar at 5 °C, with scores of 6.3 ± 0.18 compared with those obtained at 2 °C (4.2 ± 0.15).

## 4. Discussion

Information about changes in nutrition, bioactive compounds, and antioxidant activity of the blood orange cultivars can provide a comprehensive insight for assessment of the best storage period as a result of maintaining the highest fruit quality during long-term cold storage. In this paper, a comparative study of four blood orange cultivars (“Moro”, “Tarocco”, “Sanguinello”, and “Sanguine”) stored for 180 days at 2 and 5 °C was carried out.

Citrus fruit after harvest and during cold storage needs to provide energy by catabolism of organic acids and sugars as substrates for maintaining cellular metabolism. Accumulation and degradation of sugars and organic acids occur through glycolysis and the Krebs cycle. On the other hand, the reduction in organic acids and sugars in the flesh may be affected by biosynthesis and catabolism [29]. Therefore, different cellular metabolism and catabolism of organic acids and sugars concentrations in citrus fruit are crucial to assess citrus fruit postharvest life in prolonged storage. In this study, the major sugars of blood orange cultivars were sucrose, glucose, and fructose, of which levels at harvest were different depending on cultivar, but all the concentration of sugars reduced during storage at both 2 and 5 °C. Differences in initial individual sugars concentration among cultivars were probably due to the different activity of sucrose synthase and sucrose phosphate synthase or invertases [30]. In addition, sucrose metabolism depends on β-fructosidase and α-glucosidase activities, which lead to the formation of fructose and glucose, respectively [31]. Furthermore, individual sugars increased at initial sampling time and then, decreased at both temperatures. The reduction in sucrose, glucose, and fructose was probably due to senescence or sugars consumption in the respiratory process for ATP production during long-term storage [1]. Another mechanism for sugars reduction is carbohydrate transport from flesh to pericarp or carbohydrate redistribution that might be occurred in pumelo cultivars [29]. These authors hypothesized that pericarp has a direct contact with the surrounding storage air and fruit carries out cellular respiration due to adequate oxygen supply and consumes substrates by this aforementioned mechanism.

Besides sugars content, organic acids are important components of citrus fruit juice and their concentration depends on the fruit species and cultivars [31,32]. In our study, the main organic acids were citric, malic, succinic, and oxalic acids, of which concentrations were different among blood orange cultivars, although all decreased during cold storage at both temperatures. Citric and malic acids were the main organic acids in blood oranges, respectively, with concentrations being higher in “Sanguinello” and lower in “Tarocco”. The different organic acids content of cultivars, especially citric acid at initial sampling time, could be related to the H^+^-ATPase pump on the vacuolar membrane, which can provide a large influx of H^+^ within tonoplast. This proposed mechanism can accumulate additional citric acid in citrus fruit [31]. The reduction in organic acids during cold storage is probably due to their consumption as main substrates for energy production, providing carbon skeletons for the synthesis of phenolic compounds and also synthesis of sugars from organic acids [1]. In addition, the reduction in organic acids could be related to fruit senescence after prolonged storage. In this study, reduction in organic acids at 2 was greater than 5 °C and might be related to alcoholic fermentation at lower temperature [13].

Among the citrus species, blood oranges accumulate anthocyanin pigments which are considered as a quality index [1,12]. Low temperature can stimulate the synthesis of anthocyanins in blood oranges during storage [16], mainly due to activation of the phenylpropanoid pathway [17].

In this study, all cultivars had a pale red color at harvest except “Sanguinello” and TAC increased during cold storage, the enhancement of TAC at 5 °C being significantly higher than at 2 °C. Accordingly, TAC increased in “Moro”, “Tarocco”, “Sanguinello”, and “Sanguine” up to 31-fold, 14-fold, 11-fold, and 20-fold, respectively at 5 °C, while at 2 °C it remained almost unchanged. Anthocyanin synthesis in blood oranges during cold storage depends on the activation of the enzymes involved in phenylpropanoid metabolism. Therefore, activation of these enzymes can be induced by low temperatures [14]. For example, the expression of structural genes involved in the phenylpropanoid biosynthesis pathway, including PAL, CHS, DFR, and UFGT at 4 °C, was higher than in the “Tarocco” cultivar stored at 25 °C, suggesting that low temperature strongly induced the transcriptome of gene expression [14]. In addition, it has been reported that expression levels of most genes involved in the phenylpropanoid pathway were higher in blood orange than in blond ones [11]. In addition, TAC rose 500% and 19% in “Tarocco” and “Moro”, respectively, stored at 8 °C after 86 days in comparison with concentration at harvest [16]. Moreover, TAC in the “Tarocco” cultivar raised up to 87% at 4 °C after 70 days of storage in comparison with initial levels [17]. Therefore, anthocyanins can increase under moderate cold temperatures, as occurred at 5 °C for all cultivars, and especially for “Sanguinello”, which reached the maximum anthocyanins levels after 180 days plus 2 days at 20 °C, which is an 11-fold increase. In this study, cyanidin 3-glucoside and cyanidin 3-(6″-malonylglucoside) were two main anthocyanins in the four blood orange cultivars, the latter being found at higher concentrations, and both showed the same trend as TAC during storage at both temperatures for all cultivars. Previous studies suggested that metabolic pathways involved in anthocyanins biosynthesis in blood oranges could have been partially inhibited at very low temperatures, such as below 3 °C [11]. A possible explanation could be that for all cultivars, PAL activity was higher at 5 than at 2 °C, as was the anthocyanins content. In fact, a positive close relationship was found between PAL activity and anthocyanin accumulation (R^2^ = 0.65–0.79). The anthocyanins content followed the order “Sanguinello” > “Moro” > “Sanguine” > “Tarocco”, as did the PAL activity.

On the other hand, stability and degradation of the anthocyanin molecule depend on PPO and POD activities during cold storage [12]. However, in this study, POD activity was not detected in the flesh of any cultivar. In addition, PPO activity was very low for all cultivars during storage and without significant differences between the two temperatures. This low activity was probably attributable to acidic conditions due to the high content of organic acids of blood orange fruit [33].

In this study, TPC increased at both temperatures and then remained constant or decreased to the end of storage, although concentrations were higher at 5 °C. As occurred with TAC, the changes of phenolic compounds could be attributed to PAL activity since a close relationship was found (R^2^ = 0.67–0.72). Similarly, TAA increased for all cultivars and then, decreased to the end of storage, the reduction being higher at 2 °C. However, ascorbic acid content was drastically reduced over storage for all cultivars and probably did not contribute to TAA in blood oranges. These results would confirm previous reports in which there was a positive correlation between TAC and TPC with TAA [15]. Cold temperatures can induce the accumulation of reactive oxygen species (ROS) and fruits use enzymatic and non-enzymatic antioxidant systems to scavenge the ROS generated at low temperatures [34]. Antioxidant systems including CAT, APX, and SOD activities can scavenge ROS. In this study, antioxidant enzyme activities increased up to 30 or 60 days and then, decreased to the end of storage at both temperatures. In addition, non-enzymatic antioxidants, including ascorbic acid and phenolic compounds, can act as another mechanism for scavenging ROS [35]. In this study, the reduction in ascorbic acid at 2 °C was probably due to overproduction of ROS and then, ascorbic acid was used as an electron donor to neutralize ROS during storage [1]. Therefore, in blood oranges, antioxidant bioactivity involves both enzymatic and non-enzymatic systems [1,2]. Total antioxidant activity in blood oranges derives from antioxidant enzymes and bioactive compounds including total anthocyanin, total phenols, and ascorbic acid that are responsible for the antioxidant system [13,15]. In addition, CAT, APX, and SOD enzymes activities can influence the enzymatic system. However, antioxidant enzymes can influence antioxidant activity in the juice of blood orange fruit, but non-enzymatic systems including anthocyanin and total phenolics are a major part of the antioxidant activity of blood orange fruit [1]. It shows that there are relationships between cultivars with the highest content of bioactive compounds and TAA. In this study, “Sanguinello” showed this relationship with the highest TAC, TPC, and TAA.

Blood oranges are non-climacteric fruit with low metabolic activities, but biochemical changes can influence largely on fruit quality during long-term cold storage [1]. Citrus fruit taste depends on sugars and organic acids, which are reduced at higher rates compared with sugars and therefore, flavor changes after long-term storage [28]. In this study, cultivar and storage temperature affected the acceptability of blood oranges, and in many cases, subtropical crops, such as citrus fruit stored at lower than optimal temperatures, induce undesirable changes [36]. Overall, panelists gave the higher scores to “Sanguinello”, which could be attributed to a better balance between sugars and acids than the other cultivars, together with the enhancement of anthocyanins. Sugars are considered as the main soluble components in the flesh of citrus fruit and are responsible for sweetness of the juice. Since fructose and glucose are 80% and 60% sweeter than sucrose, respectively, fruit taste depends on the proportions of sucrose, glucose, and fructose [35]. Sensory evaluation revealed that the lowest edible quality for all blood orange cultivars was at 2 °C, which could be related to a fermentative metabolism that can produce some compounds that reduce the edible quality and occurrence of off-flavors [28]. For example, ethanol, acetaldehyde, furaneol, and polyvinylguaiacol are related to the off-flavors in citrus fruit at cold temperature. In addition, changes in the volatile and non-volatile components at suboptimal temperature can produce non-volatile flavor compounds that had a negative effect on citrus fruit taste, including putrescine and limonin as reported in sweet orange cultivars [37].

## 5. Conclusions

This is the first comparative study on the changes in bioactive compounds, antioxidant activity, and nutritional quality of four blood orange cultivars at different storage temperatures. For all cultivars, the temperature of 2 °C could not be appropriated for long-term storage compared with 5 °C. At this temperature, bioactive compounds such as TAC, TPC, and individual anthocyanins cyanidin 3-glucoside and cyanidin 3-(6″-malonylglucoside) were enhanced, which is consistent with the increased PAL activity. Among cultivars, “Sanguinello” was the best one, since quality parameters were better retained during the 180 days of storage. Based on these results, we propose that a moderate cold temperature after harvest can increase bioactive compounds, especially anthocyanin and phenolic compounds, which are related to human health.

## Supplementary Materials

The following are available online at https://www.mdpi.com/2076-3921/9/10/1016/s1, Figure S1: HPLC chromatograms of individual anthocyanins cyanidin 3-glucoside and cyanidin 3-(6″-malonylglucoside); Figure S2: HPLC chromatograms of individual organic acids (citric, ascorbic, malic, oxalic and succinic acids); Figure S3: HPLC chromatograms of individual sugars (sucrose, glucose and fructose); Table S1: Precision and recovery of the HPLC for determination of individual anthocyanins; Table S2: Precision and recovery of the HPLC for determination of individual sugars; Table S3: Precision and recovery of the HPLC for determination of individual organic acids.
