# Supplementary material for: Changes in Bioactive Compounds, Antioxidant Activity, and Nutritional Quality of Blood Orange Cultivars at Different Storage Temperatures
Source: Antioxidants (Basel). 2020 Oct 20;9(10):1016. doi: 10.3390/antiox9101016 (PMC7589990; doi:10.3390/antiox9101016)
Supplement: Supplementary file 1 [file antioxidants-09-01016-s001.pdf]

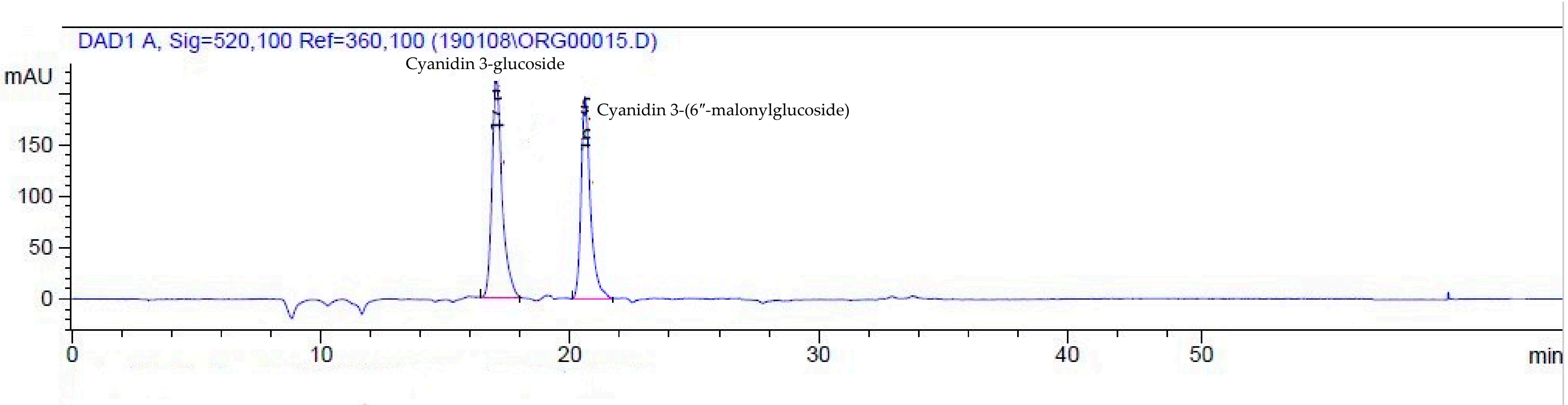


Figure S1. HPLC chromatograms of individual anthocyanins cyanidin 3-glucoside and cyanidin 3-(6″-malonylglucoside).

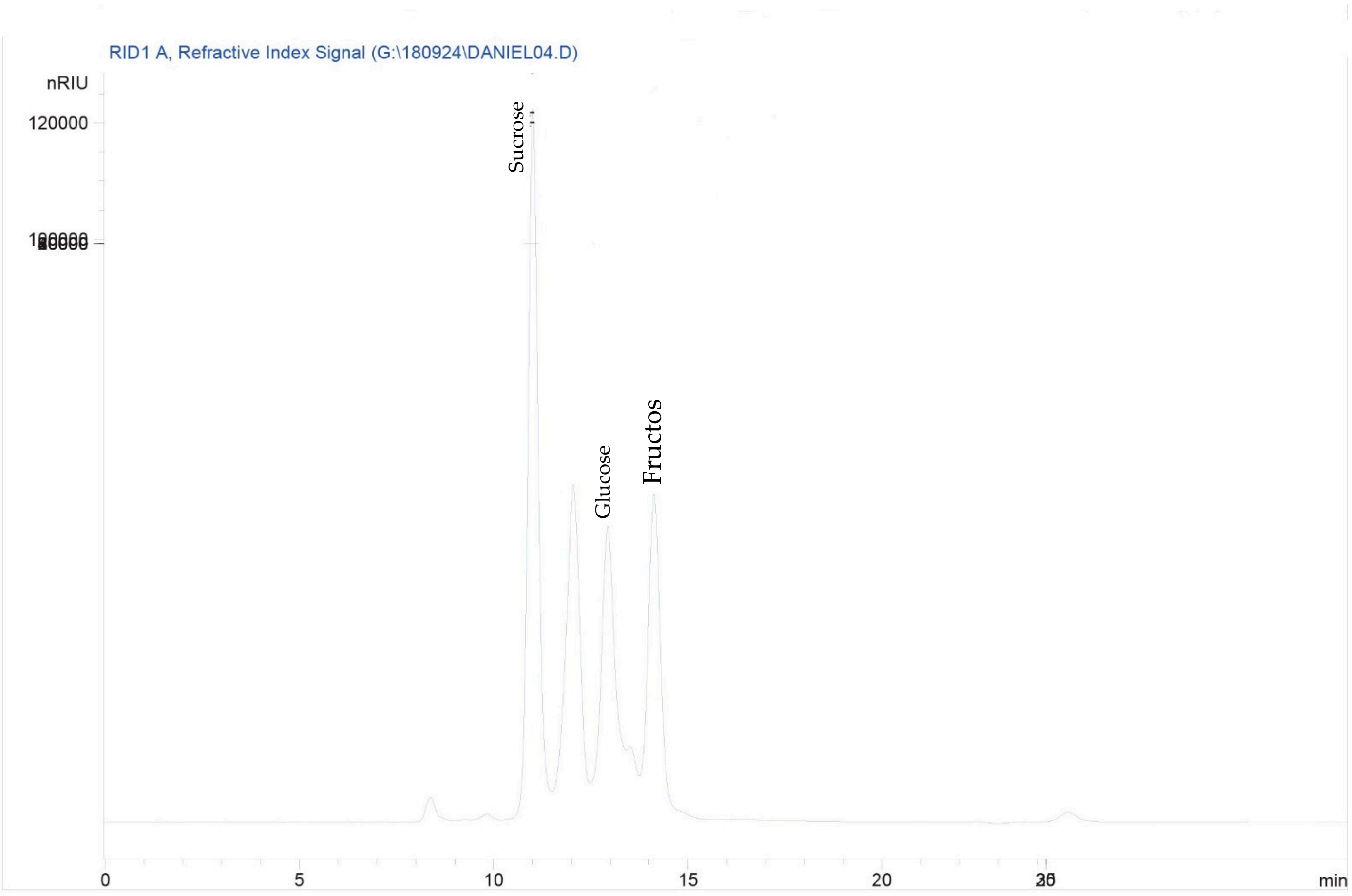


Figure S2. HPLC chromatograms of individual sugars (sucrose, glucose and fructose).

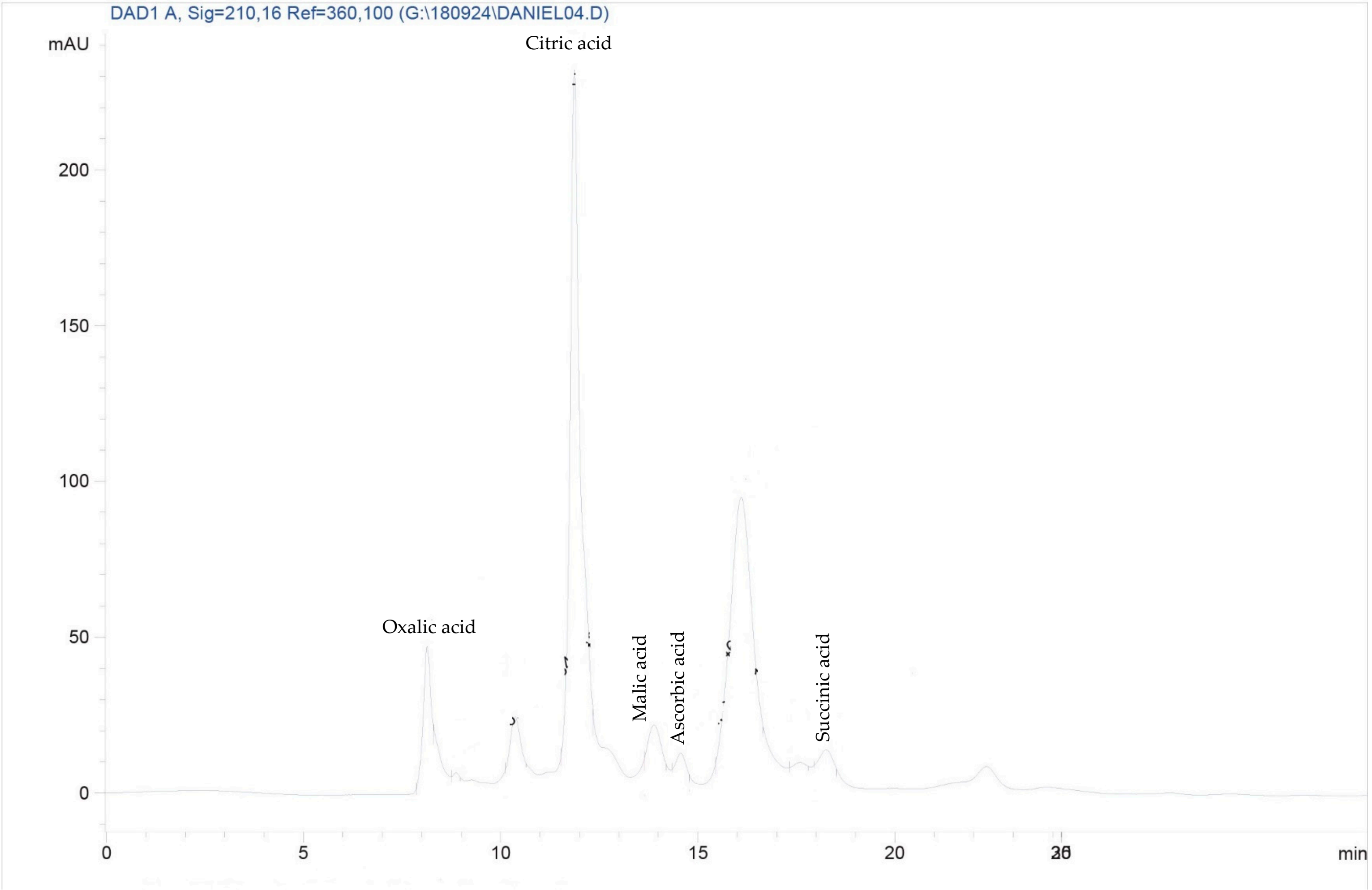


Figure S3. HPLC chromatograms of individual organic acids (citric, ascorbic, malic, oxalic and succinic acids).

**Table S1. Precision and recovery of the HPLC for determination of individual anthocyanins.**

| Individual anthocyanins | Parameters | Sample No. | Concentration (μg/mL) | Mean | Standard deviation (SD) | Precision (% RSD) | Recovery (%) |
|---|---|---|---|---|---|---|---|
| Cyanidin 3-glucoside | Intra-day (n=3) | 1 | 0.663 | 0.659 | 0.022 | 3.338 | 99.4 |
| | | 2 | 0.661 | 0.657 | 0.017 | 2.587 | 99.39 |
| | | 3 | 0.689 | 0.685 | 0.046 | 6.715 | 99.42 |
| | Inter-day (n=3) | 1 | 0.663 | 0.665 | 0.031 | 4.661 | 100.3 |
| | | 2 | 0.661 | 0.665 | 0.016 | 2.406 | 100.61 |
| | | 3 | 0.689 | 0.675 | 0.042 | 6.222 | 97.97 |
| Cyanidin 3-(6″-malonylglucoside) | Intra-day (n=3) | 1 | 7.912 | 7.909 | 0.441 | 5.575 | 99.96 |
| | | 2 | 7.901 | 7.894 | 0.353 | 4.471 | 99.91 |
| | | 3 | 8.238 | 8.221 | 0.226 | 2.749 | 99.79 |
| | Inter-day (n=3) | 1 | 7.912 | 7.911 | 0.338 | 4.272 | 99.99 |
| | | 2 | 7.901 | 7.989 | 0.447 | 5.595 | 101.11 |
| | | 3 | 8.238 | 8.229 | 0.417 | 5.067 | 99.89 |

**Table S2. Precision and recovery of the HPLC for determination of individual sugars.**

| Individual sugars | Parameters | Sample No. | Concentration (μg/mL) | Mean | Standard deviation (SD) | Precision (% RSD) | Recovery (%) |
|---|---|---|---|---|---|---|---|
| Sucrose | Intra-day (n=3) | 1 | 4.842 | 4.822 | 0.076 | 1.576 | 99.59 |
| | | 2 | 4.843 | 4.837 | 0.106 | 2.191 | 99.88 |
| | | 3 | 4.601 | 4.543 | 0.089 | 1.959 | 98.74 |
| | Inter-day (n=3) | 1 | 4.842 | 4.873 | 0.112 | 2.298 | 100.64 |
| | | 2 | 4.843 | 4.848 | 0.108 | 2.227 | 100.1 |
| | | 3 | 4.601 | 4.499 | 0.048 | 1.066 | 97.78 |
| Glucose | Intra-day (n=3) | 1 | 2.152 | 2.144 | 0.088 | 4.104 | 99.63 |
| | | 2 | 2.202 | 2.191 | 0.094 | 4.29 | 99.50 |
| | | 3 | 2.165 | 2.157 | 0.065 | 3.013 | 99.63 |
| | Inter-day (n=3) | 1 | 2.152 | 2.126 | 0.075 | 3.527 | 98.79 |
| | | 2 | 2.202 | 2.169 | 0.068 | 3.135 | 98.50 |
| | | 3 | 2.165 | 2.136 | 0.084 | 3.932 | 98.66 |
| Fructose | Intra-day (n=3) | 1 | 2.622 | 2.608 | 0.052 | 1.993 | 99.47 |
| | | 2 | 2.583 | 2.573 | 0.027 | 1.049 | 99.61 |
| | | 3 | 2.635 | 2.628 | 0.031 | 1.179 | 99.73 |
| | Inter-day (n=3) | 1 | 2.622 | 2.592 | 0.055 | 2.121 | 98.86 |
| | | 2 | 2.583 | 2.532 | 0.045 | 1.777 | 98.03 |
| | | 3 | 2.635 | 2.611 | 0.028 | 1.072 | 99.09 |

**Table S3. Precision and recovery of the HPLC for determination of individual organic acids.**

| Individual sugars | Parameters | Sample No. | Concentration (μg/mL) | Mean | Standard deviation (SD) | Precision (% RSD) | Recovery (%) |
|---|---|---|---|---|---|---|---|
| Oxalic acid | Intra-day (n=3) | 1 | 0.375 | 0.361 | 0.018 | 4.986 | 96.27 |
| | | 2 | 0.365 | 0.378 | 0.021 | 5.555 | 103.56 |
| | | 3 | 0.377 | 0.364 | 0.025 | 6.868 | 96.55 |
| | Inter-day (n=3) | 1 | 0.375 | 0.359 | 0.019 | 5.292 | 95.73 |
| | | 2 | 0.365 | 0.359 | 0.025 | 6.963 | 98.36 |
| | | 3 | 0.377 | 0.369 | 0.011 | 2.981 | 97.88 |
| Citric acid | Intra-day (n=3) | 1 | 0.477 | 0.462 | 0.015 | 3.246 | 96.86 |
| | | 2 | 0.463 | 0.451 | 0.018 | 3.991 | 97.41 |
| | | 3 | 0.469 | 0.465 | 0.016 | 3.441 | 99.15 |
| | Inter-day (n=3) | 1 | 0.477 | 0.459 | 0.014 | 3.051 | 96.23 |
| | | 2 | 0.463 | 0.456 | 0.015 | 3.289 | 98.49 |
| | | 3 | 0.469 | 0.454 | 0.014 | 3.083 | 96.80 |
| Malic acid | Intra-day (n=3) | 1 | 38.66 | 38.38 | 1.877 | 4.89 | 99.28 |
| | | 2 | 37.54 | 37.08 | 1.631 | 4.398 | 98.77 |
| | | 3 | 37.43 | 37.29 | 1.71 | 4.585 | 99.63 |
| | Inter-day (n=3) | 1 | 38.66 | 37.65 | 1.644 | 4.366 | 97.39 |
| | | 2 | 37.54 | 36.58 | 1.559 | 4.261 | 97.44 |
| | | 3 | 37.43 | 36.91 | 1.498 | 4.058 | 98.61 |
| Ascorbic acid | Intra-day (n=3) | 1 | 15.56 | 15.27 | 0.546 | 3.575 | 98.14 |
| | | 2 | 15.63 | 15.55 | 0.525 | 3.376 | 99.49 |
| | | 3 | 15.54 | 15.41 | 0.505 | 3.277 | 99.16 |
| | Inter-day (n=3) | 1 | 15.56 | 15.22 | 0.592 | 3.889 | 97.81 |
| | | 2 | 15.63 | 15.19 | 0.491 | 3.232 | 97.18 |
| | | 3 | 15.54 | 15.46 | 0.515 | 3.331 | 99.49 |
| Succinic acid | Intra-day (n=3) | 1 | 37.96 | 37.11 | 1.659 | 4.47 | 97.76 |
| | | 2 | 38.93 | 38.63 | 1.397 | 3.616 | 99.23 |
| | | 3 | 38.88 | 38.22 | 1.232 | 3.223 | 98.30 |
| | Inter-day (n=3) | 1 | 37.96 | 36.97 | 1.575 | 4.26 | 97.39 |
| | | 2 | 38.93 | 37.84 | 1.681 | 4.442 | 97.20 |
| | | 3 | 38.88 | 36.73 | 1.768 | 4.813 | 94.47 |
